# Analytical Ultracentrifugation to Assess the Quality of LNP-mRNA Therapeutics

**DOI:** 10.3390/ijms25115718

**Published:** 2024-05-24

**Authors:** Giuditta Guerrini, Dora Mehn, Diletta Scaccabarozzi, Sabrina Gioria, Luigi Calzolai

**Affiliations:** European Commission, Joint Research Centre (JRC), 21027 Ispra, Italy; giuditta.guerrini@ec.europa.eu (G.G.); dora.mehn@ec.europa.eu (D.M.); diletta.scaccabarozzi@ec.europa.eu (D.S.); sabrina.gioria@ec.europa.eu (S.G.)

**Keywords:** LNP-mRNA, therapeutics, analytical ultracentrifugation (AUC), vaccine, quality, free RNA

## Abstract

The approval of safe and effective LNP-mRNA vaccines during the SARS-CoV-2 pandemic is catalyzing the development of the next generation of mRNA therapeutics. Proper characterization methods are crucial for assessing the quality and efficacy of these complex formulations. Here, we show that analytical ultracentrifugation (AUC) can measure, simultaneously and without any sample preparation step, the sedimentation coefficients of both the LNP-mRNA formulation and the mRNA molecules. This allows measuring several quality attributes, such as particle size distribution, encapsulation efficiency and density of the formulation. The technique can also be applied to study the stability of the formulation under stress conditions and different buffers.

## 1. Introduction

Lipid Nanoparticles-encapsulated mRNA (LNPs-mRNA) have played a pivotal role in the global response to the Coronavirus disease 2019 (COVID-19) pandemic, with proven safety and efficacy, and are now at various stages of development against a variety of other infectious diseases or studied for cell and gene therapies [1,2,3,4,5,6].

LNP-mRNA formulations used in Severe acute respiratory syndrome coronavirus 2 (SARS-CoV-2) vaccines contain four different lipids and the cargo, a ribonucleic molecule, encoding a protein intended to elicit an immune response, in case of vaccines, or to treat aberrantly expressed gene(s), in case of genetic diseases.

Various studies demonstrated that the proper assembly and stability of LNPs are particularly crucial for their safety and efficacy [7,8,9]. We have also previously published a characterization cascade suggested to address key challenges in the preclinical characterization and identify potential pitfalls during the LNPs-mRNA synthesis [10].

The implementation of a structured characterization strategy contributes to improve RNA therapeutics quality and reduces development times, a critical aspect, especially in response to emerging pathogen outbreaks [10,11].

The size of the LNPs-mRNA particles and the amount of encapsulated mRNA are two critical quality attributes strongly related to in vitro potency. Based on recommendations from the Food and Drug Administration (FDA) and International Council for Harmonisation of Technical Requirements for Registration of Pharmaceuticals for Human Use (ICH) Q2R2 guidance documents applicable to LNP-mRNA formulation, for the validation of the measurement of complex quality attributes, a combination of orthogonal techniques that ideally applies different measurement principles is recommended [12,13].

Analytical Ultracentrifugation (AUC) has been applied as a sizing method for macromolecules [14,15,16,17,18] although the technique has broader applications including nanomaterials [19,20,21,22] and liposomes [23]. It separates particles based on their sedimentation velocity and the resulting sedimentation coefficient distributions are a function of the mass, density and shape of each particle population present in the sample.

AUC has been already applied to study LNPs formulations [24,25,26]. LNPs-mRNA have densities lower than 1 (typically between 0.9 and 1 g/mL), thus floating, rather than sedimenting, during the centrifugal separation. Existing software can handle such systems: data analysis of floating particles gives distributions in the negative sedimentation coefficient regime [24]. This can be transformed to size distribution if the density of the particles is known or calculated by running several measurements at various liquid densities [7,26]. Up to now, data analysis is performed considering either only the positive range of sedimentation coefficients (sedimenting particles) or the negative range (floating particles). This is limiting, in fact LNP-mRNA formulation contains components with densities smaller than 1 (all the lipids) and higher than 1 (the mRNA) and the current type of analysis cannot detect the presence of both types of components in a single analysis. Here, we show that it is possible to simultaneously detect components floating and sedimenting within a single AUC experiment.

This opens up the capacity of AUC to measure the integrity of the LNP-mRNA systems and, simultaneously, the eventual amount of free mRNA, thus providing valuable information on the quality of the starting material, the encapsulation of the mRNA molecules, and the possibility to follow changes caused by stressors such as temperature and storage. Furthermore, this technique does not require any sample preparation apart from a dilution in a suitable buffer, which allows the running of measurements under physiological conditions.

## 2. Results and Discussion

LNP-mRNA samples were synthesized starting from a mixture of lipids (ionizable lipid, cholesterol, DSPC and PEG-lipid) in a relative molar ratio of 50:37.5:10:2.5 (very similar to the lipid composition of the approved LNP-mRNA vaccines) and encapsulating mRNA expressing the firefly luciferase enzyme (FLUC mRNA).

The LNP-mRNA formulations were characterized with the “minimal characterization approach” [7] by measuring the size and polydispersity (PDI) with DLS and the total mRNA content by the RiboGreen assay. Table 1 shows that the samples have sizes in the range of 70 to 110 nm and are well monodispersed, with PDI values smaller than 0.1. All samples have encapsulation efficiency larger than 95%, except for LNP-2 (74%).

The AUC data were acquired at moderate rotational speed (10,000 rpm) with the UV absorbance detector set at a wavelength specific for the ribonucleic bases (260 nm). By modifying the data processing parameters (i.e., allowing both positive and negative sedimentation coefficients in the fitting routine, see Materials section for details), we were able to detect the presence of both sedimenting and floating objects in a given sample.

**Figure 1:**
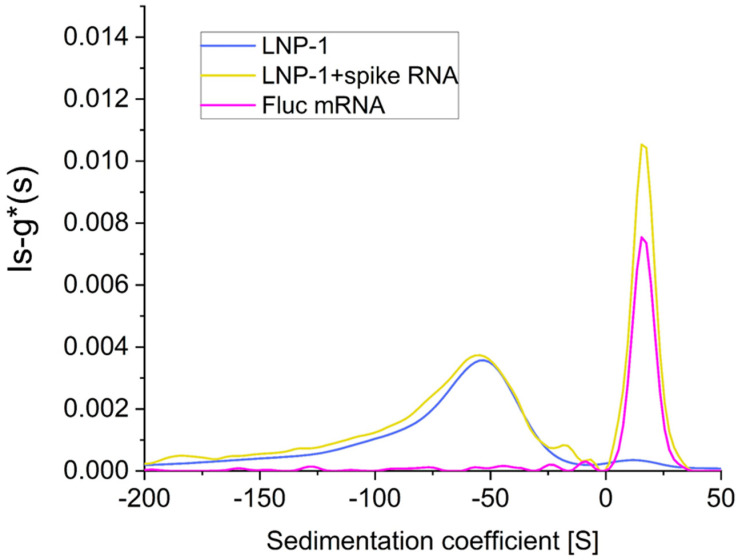
Sedimentation coefficient distribution of LNP formulation (LNP-1) alone (blue) or spiked with FLuc mRNA (yellow) and FLuc mRNA alone (pink).

Figure 1 shows the distribution of sedimentation coefficients for a sample containing the LNP formulation (LNP-1) spiked with free FLuc mRNA (yellow). The data show the presence of two peaks: one at around −50 S (floating) and one at around +18 S (sedimenting). Comparing these results with samples containing only LNP-1 (blue) and only FLuc mRNA (pink), it is possible to assign the −50 S peak to LNP-mRNA and the +18 S peak to the free mRNA. These values are comparable to the values reported in literature for LNP-mRNA [12] and measured in our experiments for the free mRNA (Figure 1, pink). Fitting the data for a free mRNA sample with the continuous c(s,ff0) model gives a molecular weight of around 520 KDa, in good agreement with the nominal molecular mass of 540 KDa.

As shown above, it is possible to simultaneously measure the sedimentation coefficient of LNP-mRNA components that float and sediment in the same experiment within a single AUC run. Results also indicate that in this particular case, the free mRNA added afterward to the final product has no interaction with the LNP-mRNA particles: in fact, the sedimentation coefficient (and the area of the peak) of the floating component is very similar for the LNP-1 alone and for the LNP-1 spiked with FLuc mRNA (Figure 1).

**Figure 2:**
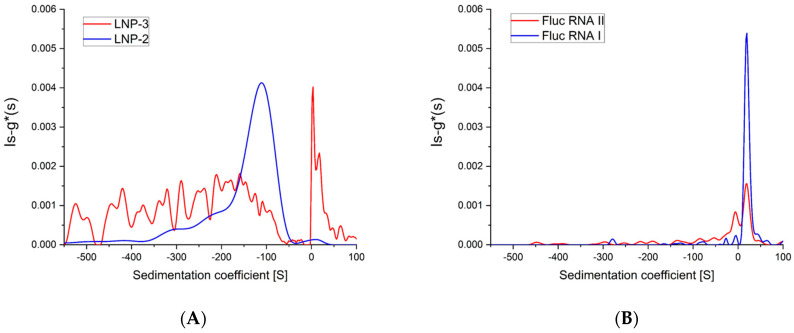
Sedimentation coefficient distribution of (**A**) LNP-2 (blue) and LNP-3 (red) formulations, (**B**) FLuc mRNA-I (blue) and FLuc mRNA-II (red).

This method is also able to quickly detect sample-to-sample variability, which may not be identified by other routinely applied techniques. Figure 2B (right side) shows the sedimentation coefficient distribution of samples LNP-2 and LNP-3. It is immediately clear from the data that sample LNP-3 is quite different from LNP-1 and LNP-2.

While LNP-2 (Figure 2A, blue line) is floating (peak at around −100 S), LNP-3 (Figure 2A, red line) is sedimenting (main peak at around +10 S) in addition to a broad and noisy floating component. This suggests that LNP-3 particles are not well formed, quite heterogeneous and with a significant amount of non-encapsulated mRNA. The initial characterization with DLS analysis and encapsulation efficiency (Table 1) demonstrated some indication that LNP-3 is different from LNP-1 and LNP-2 but did not reach the level information provided by AUC data. The shift of the sedimentation coefficient distribution to more negative sedimentation coefficient (s) values compared to LNP1 (in case of both formulations) might be correlated to the difference in encapsulation efficiency. Particles containing less mRNA have lower density and tend to float faster [7].

The two samples LNP-2 and LNP-3 have been synthesized using two different aliquots of the same FLUC mRNA batch. Figure 2B clearly shows that the mRNA-II starting material (used for the LNP-3 formulation), although presenting a similar sedimentation coefficient for the main component, has a different sedimentation coefficient distribution compared to mRNA-I used for the production of LNP-1 and LNP-2. The presence of several peaks with positive sedimentation values (Figure 2B, red line) suggests a possible fragmentation of the starting material or the presence of significant product contamination.

**Figure 3:**
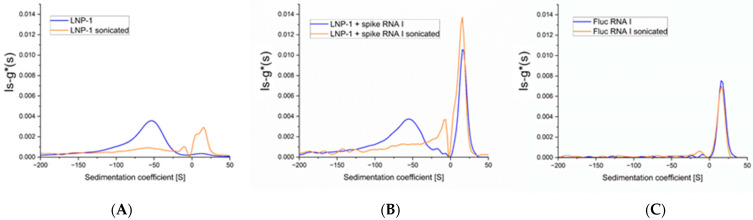
Sedimentation coefficient distribution after sonication (orange) compared to original sample (blue). (**A**) LNP-1 formulation, (**B**) LNP-1 spiked with FLuc mRNA, (**C**) FLuc mRNA alone.

To assess the capacity of AUC to detect changes in the LNP-mRNA particle formulations, we disrupted the LNP-1 sample by controlled sonication. The use of a vial tweeter sonicator allows us to finely control and reproduce the amount of energy while avoiding possible contamination. Figure 3A shows that, after sonication, the peak at −50 S disappears (or is severely reduced and broadened), while new peaks with positive sedimentation values appear at +18 S and a shoulder at +10 S. The peak at +18 S is easily assigned to free mRNA released from the particles. The shoulder at +10 S is more difficult to attribute: it could be due to mRNA fragments generated during the sonication process, but sonication of free mRNA (under the same experimental conditions) fails to produce any mRNA fragment (Figure 3C). Our hypothesis is that the shoulder at +10 S could be the signature of chemical adducts between the lipid components and the mRNA molecules [27].

**Figure 4:**
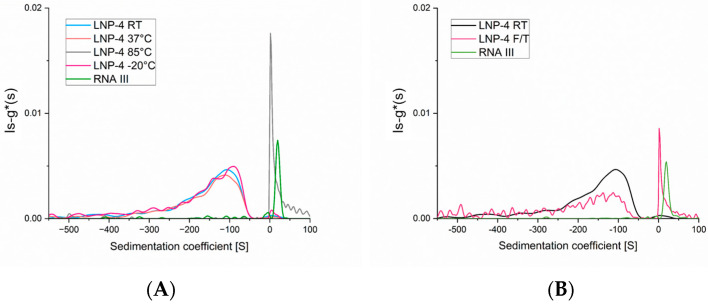
Sedimentation coefficient distribution after thermal stress of LNP-4 formulation. (**A**) LNP-4 RT (blue), +37 °C (orange), +85 °C (grey), −20 °C (pink) and FLuc mRNA alone (green). (**B**) LNP-4 RT (blue), LNP-4 F/T (black) and FLuc mRNA alone (green).

We then subjected the LNP-mRNA formulation (LNP-4) to thermal stress by storing aliquots of the sample at different temperatures (room temperature, +37 °C, +85 °C and −20 °C) for 15 min, and then, performing AUC measurements at 20 °C. Figure 4A shows that samples kept at +37 °C and −20 °C are essentially the same as the reference sample kept at room temperature, while the sample heated at +85 °C presents only a broad peak at positive s values, probably due to the release of mRNA. Comparing the samples at 37 °C and 85 °C with AF4-DLS measurements (Supporting Information, Figure S1) show that the sample kept at high temperature has a diameter significantly larger than that at 37 °C (180 nm versus 70 nm) and a much broader particle size distribution.

Due to the excellent results of AUC being able to detect and measure disruption of the particle formulation, we subjected an LNP-mRNA sample to freeze/thaw (F/T) stress. Samples were frozen at −80 °C and then thawed at room temperature; each F/T cycle was repeated four times. Figure 4B shows that after stressing the sample by F/T (mimicking a possible scenario that can happen in the cold chain of LNP-mRNA therapeutics), the LNP-mRNA negative peak is much less intense and new peaks of sedimenting species appear at +10S with a shoulder at +18S. This clearly indicates that some of the nanoparticles are disrupted, and free mRNA and probably some mRNA–lipid adducts are released.

AUC is a robust method for measuring the particle size distribution of LNP-mRNA formulations, underpinned by extensive scientific literature [20,28,29] and existing protocols [25,27,28,29,30]. The application of the technique requires quite substantial instrument and expertise investment, but very limited sample preparation and relatively small efforts in terms of time needed for parameter optimization and data analysis.

AUC can detect both the floating and the sedimenting fractions of any LNP-mRNA formulation. As described earlier and supported also by standard methods, it allows determination of particle densities and, using these density values, the size distribution profiling of a sample [7,31,32,33]. Moreover, as it is shown here, the technique can also discriminate the mRNA encapsulated in the LNP formulations from the non-encapsulated free mRNA molecules or mRNA–lipid adducts.

## 3. Materials and Methods

Pre-made lipid mixture (GenVoy-ILM™) containing ionizable lipid, cholesterol, DSPC and PEG-lipid in a relative molar ratio of 50:37.5:10:2.5 was purchased from Precision NanoSystems (Vancouver, BC, Canada). CleanCap^®^ FLuc mRNA modified in the Uridine bases (5moU) was purchased from Tebu-Bio (Cat 040L-7202, Roskilde, Denmark). The same batch was divided in aliquots and stored at −80 °C. Aliquot-II has been frozen/thawed multiple times before being used for encapsulation in LNP.

### 3.1. LNP Formulation

LNPs were synthesized using the Nanoassemblr Ignite^®^ instrument (Precision NanoSystems, Vancouver, BC, Canada) according to the manufacturer’s instructions. Briefly, GenVoy-ILM™ lipids mixture in ethanol were mixed with CleanCapR FLUC mRNA suspended in acidic buffer, using the NxGen microfluidic cartridge. The nitrogen to phosphate ratio (N/P) between the ionizable lipid and the mRNA was maintained at 4, while the flow rate and the aqueous to organic ratio were respectively FR: 12 mL/min and FRR: 3:1 (RNA/lipid). For all formulations, the initial and final waste volumes were set to 0.45 and 0.05 mL, respectively. After the formulation was processed in the micromixer, residues of ethanol and acidic buffer were removed by dialysis with a centrifugation step in PBS, at 2000 rpm, 4 °C, for 2 h using 10 kDa molecular weight cut-off dialysis Amicon filter (EMD Millipore, Billerica, MA, USA). The formulations were finally sterilized through a 0.22 μm filter and stored at 4 °C until use.

### 3.2. Stress Conditions

Sonication of samples was performed in 1.5 mL Eppendorf tube (Eppendorf, Hamburg, Germany), for 1’ at 75% intensity, pulse every 0.5 s by vial tweeter sonicator (UIS250v, Hielscher, Teltow, Germany). Temperature stresses of +37 °C and +85 °C were achieved in an Eppendorf thermomixer for 15’ while the −20 °C sample was put in a 1.5 mL Eppendorf tube and frozen for 15’. Freeze and thaw stress condition was obtained by repeating 4 cycles of freezing (−80 °C) and thawing in a 1.5 mL Eppendorf tube).

### 3.3. AUC

To analyze the sedimentation coefficient distribution of the LNPs-mRNA, sedimentation velocity-type experiments were performed using a Beckman Coulter Proteomlab XL-I analytical ultracentrifuge equipped with an 8 holes rotor. Samples were maintained at 4 °C for short storage then run after 30’ of room temperature equilibration on the bench if not otherwise specified. Samples were diluted around 40 times in PBS (, Gibco by Thermo Fisher Scientific, Waltham, MA, USA) to a final volume of 380 µL. Samples underwent temperature stress, sonication or were spiked with RNA before the loading in double-sector cells with sapphire windows. PBS was used as reference at a volume of 390 µL. Interference optics and absorbance 260/220 nm were applied to register the change in refractive index difference and absorbance at 10.000 rpm rotational speed at a nominal temperature of +20 °C.

### 3.4. Calculations and Model Fits

Sedimentation coefficient (S) distributions of the molecules were determined using the ls-g*(s) model of Sedfit choosing a range from −600 to +200 Svedberg for the fit, a linear grid with resolution of 400.

### 3.5. Ribogreen Assay

The total mRNA content of the LNP-mRNA formulations were measured by the Quant-iT RiboGreen assay (RiboGreen^®^ RNA reagent kit, Molecular Probes Cat R11490, Thermo Fisher Scientific, Waltham, MA, USA). Samples were diluted in 1 × TE buffer, or in 1 × TE buffer containing 0.5% (*v*/*v*) Triton X-100 (Sigma Aldrich Burlington, MA, USA) and run in duplicates. Standard solutions were prepared in 1× TE containing 0.5% (*v*/*v*) Triton X-100 to account for any variation in fluorescence. The assay was performed according to the manufacturer’s recommendation and using the high RNA Quantitation range (25–1000 ng/mL) calibration curve.

The fluorescence signal was measured by the EnSpire^®^ Multimode plate reader (Perkin Elmer, Shelton, CT, USA) using an excitation at 485 nm and emission at 528 nm. mRNA encapsulated was expressed as % of the total amount of mRNA.

## 4. Conclusions

AUC is a robust method for measuring the particle size distribution of LNP-mRNA formulations, underpinned by extensive scientific literature [14,19,20] and existing protocols [25,30]. The application of the technique requires quite substantial instrument and expertise investment, but very limited sample preparation and relatively small efforts in terms of time needed for parameter optimization and data analysis.

AUC is able to detect not only the floating but also the sedimenting fractions in LNP-mRNA formulations. This opens new perspectives in the application of the technique as it could be used not only for size distribution profiling of LNPs, but also to reveal the presence of sedimenting sub-population(s) in the sample and to discriminate the mRNA encapsulated in the LNP formulations from the non-encapsulated free mRNA molecules, or possibly, mRNA–lipid adducts.

The technique requires a relatively low amount of sample and no sample preparation (apart from dilution step). This is extremely important as it avoids any potential bias in the results that could be introduced by ultrafiltration steps, RNA extraction, or particle disruption that are needed when using other analytical techniques. The simplicity of the sample preparation and the specific detection of the signal from the RNA molecule (using the UV detector at 260 nm) also allow the characterization of LNP-mRNA in physiologically relevant media [34].

These results show that AUC is a very useful technique for the characterization of LNP-mRNA formulations and provide an additional technique for the quality assessment of this class of advanced therapeutics.

## Figures and Tables

**Table 1:** LNP-mRNA formulations, mRNA cargo, zeta average size (Z av), PDI and encapsulation efficiency.

Sample	mRNA	Z av (d,nm)	PDI	Encapsulation Efficiency (%)
LNP-1	I	86	0.07	97
LNP-2	I	69	0.08	95
LNP-3	II	110	0.09	74
LNP-4	III	79	0.08	99

## Data Availability

The data presented in this study are available on request from the corresponding author.

## Supplementary Materials

The following supporting information can be downloaded at: https://www.mdpi.com/article/10.3390/ijms25115718/s1.
