# Supplementary material for: Analytical Ultracentrifugation to Assess the Quality of LNP-mRNA Therapeutics
Source: Int J Mol Sci. 2024 May 24;25(11):5718. doi: 10.3390/ijms25115718 (PMC11171944; doi:10.3390/ijms25115718)
Supplement: Supplementary file 1 [file ijms-25-05718-s001.zip › ijms-2960390-supplementary.pdf]

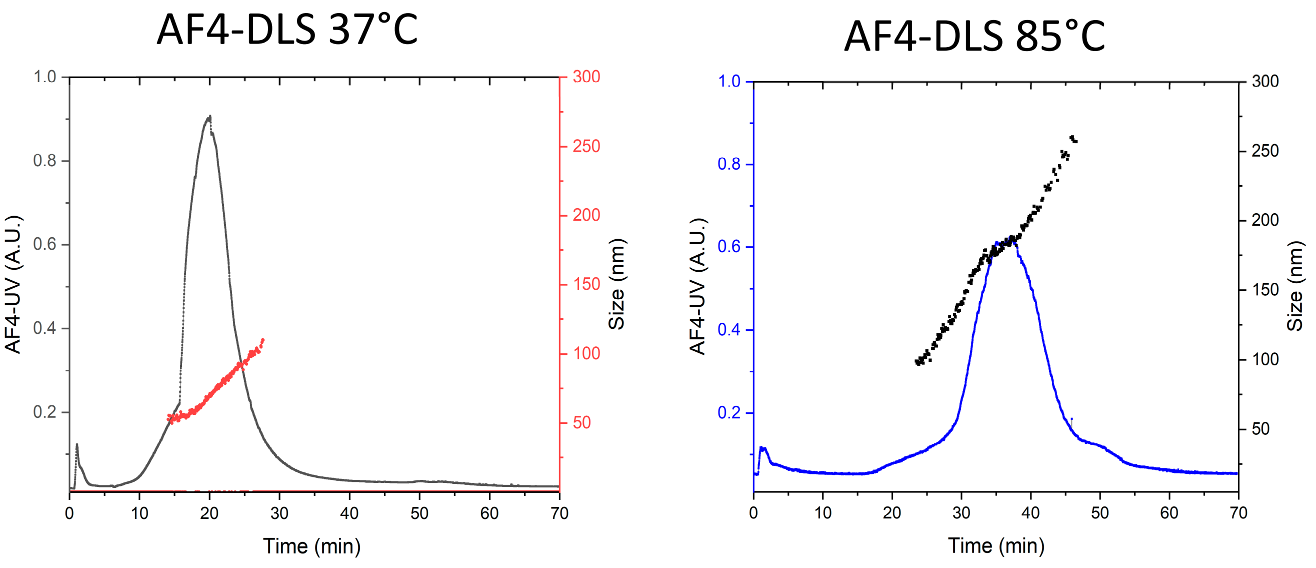

Figure S1. Asymmetric flow field flow fractionation (AF4) with online dynamic light scattering (DLS) detector of LNP-mRNA formulations kept at different temperatures. Left: sample kept at 37°C; AF4-UV fractogram black curve, size (diameter in nm) red dots and red y-axis. Right: sample kept at 85°C; AF4-UV fractogram blue curve, size (diameter in nm) black dots and black y-axis.

Aliquots of LNP-mRNA formulation (size by DLS 79 nm; encapsulation efficiency 99%) were kept for 15 minutes (in an Eppendorf thermomixer) at 37°C and 85°C. AF4-DLS measurements were collected with a Postnova Analytics AF2000 asymmetric flow field fractionation, coupled online to a Malvern ZS DLS instrument, using a Postnova frit inlet column with a 10 KDa regenerated cellulose membrane. For each measurement 20 µL of sample was injected in the column using PBS buffer as mobile phase. Separation was achieved with a flow rate of 0.5 mL/min and a cross flow program starting with 1mL/min with exponential decay to 0 mL/min in 60 min. UV detector was set at 260 nm.
